# A preliminary study on the combined assessment of 25-hydroxyvitamin D and thyroid function for predicting diabetic foot risk and amputation in type 2 diabetes

**DOI:** 10.3389/fendo.2026.1862214

**Published:** 2026-06-15

**Authors:** Dan Xiong, Yuanfang Jiang, Zhengxia Yu, Yaqin Zhong, Ke Peng, Xianglong Xia, Youqi Zhang, Lijun Liu, Weixia Peng

**Affiliations:** 1 Department of Endocrinology, Yiyang Central Hospital, Yiyang, Hunan, China; 2 Department of Critical Care Medicine, Yiyang Central Hospital, Yiyang, Hunan, China; 3 Postgraduate Collaborative Training Base, University of South China, Hengyang, Hunan, China; 4 Yiyang Central Hospital Affiliated to Hunan University of Chinese Medicine, Yiyang, Hunan, China

**Keywords:** 25-hydroxyvitamin D, amputation, euthyroid sick syndrome, thyroid function, Type 2 diabetic foot

## Abstract

**Objective:**

To examine the disparities in 25-hydroxyvitamin D (25OH-vitD) levels and thyroid function between patients with type 2 diabetic foot (DF) and those with type 2 diabetes(T2DM), and to evaluate the predictive value of 25OH-vitD combined with thyroid function for DF and its impact on amputation.

**Methods:**

A total of 79 patients with DF and 79 patients with T2DM were retrospectively enrolled. Data on general information, 25OH-vitD, FT3, FT4, TSH, and other clinical parameters were collected. Statistical analysis was performed using SPSS 27.0.

**Results:**

(1) Compared with T2DM, DF patients had significantly lower serum 25OH-vitD and FT3 levels, and higher prevalence of 25OH-vitD deficiency and euthyroid sick syndrome (ESS). (2) Multivariate logistic regression showed that the deficiency group of 25OH-vitD (<20 ng/mL) had a significantly higher risk of DF (OR = 3.539); the insufficiency group (20–30 ng/mL) showed no significant difference. FT3 was an independent protective factor (OR = 0.493). (3) ROC analysis showed that the AUC for 25OH-vitD alone was 0.620, for FT3 alone was 0.663, and for the combined predicted probability was 0.701. (4) In DF patients, 25OH-vitD was negatively correlated with disease duration and CRP. (5) In DF patients, the ESS group had lower 25OH-vitD levels than the euthyroid group. The amputation rate in the ESS group was 27.3%, significantly higher than that in the euthyroid group (8.8%). The prevalence of ESS in the major amputation group (75%) was higher than that in the minor amputation group (42.9%) and the non-amputation group (23.5%). One-way ANOVA showed significant differences in CRP and TSH among the three amputation groups. *Post-hoc* analysis indicated that patients with major amputation had significantly higher CRP and TSH levels compared to non− amputation patients. FT3 and 25OH-vitD showed a decreasing trend across the three groups (FT3: P = 0.08, 25OH-vitD: P = 0.13).

**Conclusion:**

In this pilot study, patients with diabetic foot had a higher prevalence of 25OHvitD deficiency and ESS. Combined assessment of FT3 and 25OHvitD showed a trend toward improved prediction of diabetic foot, suggesting the potential value of these markers in evaluating disease severity and prognosis.

## 1 Introduction

Type 2 diabetes foot is a prevalent and severe chronic complication among individuals with type 2 diabetes. Clinical manifestations include foot ulcers, infections, pain, and walking difficulties, with potential progression to amputation if not effectively treated in a timely manner. Epidemiological data reveal a high amputation rate of 19.03% among diabetes foot patients, with an annual mortality rate of 16.26 (1), significantly threatening patients’ quality of life and overall safety. Currently, the severity assessment of diabetic foot disease primarily relies on clinical classifications such as the Wagner grading system. However, these methods are largely based on local wound manifestations and do not fully integrate systemic metabolic and endocrine indicators, limiting their predictive value for amputation risk.

Current research suggests an association between vitamin D and the risk of various diseases, with vitamin D deficiency linked to type 2 diabetes, its microvascular complications, and peripheral neuropathy (2). However, studies on vitamin D in diabetic foot remain limited. Thyroid dysfunction is also common in diabetic patients and is associated with diabetic vascular complications.

To date, few studies have jointly evaluated the impact of vitamin D levels and thyroid function on diabetic foot disease. Among patients with type 2 diabetic foot, euthyroid sick syndrome (ESS) is the most common thyroid dysfunction, and ESS has been shown to indicate the severity of diabetic foot disease (3). However, whether ESS is associated with the risk of amputation in diabetic foot remains unclear.This retrospective study aimed to: (1) compare the differences in 25OH-vitD levels, thyroid function, and ESS prevalence between patients with type 2 diabetic foot and those with uncomplicated type 2 diabetes; (2) evaluate the predictive value of combining thyroid function and vitamin D levels for type 2 diabetic foot; and (3) explore the relationships between amputation and serum 25OH-vitD, ESS, and thyroid function in patients with type 2 diabetic foot.

## 2 Subjects and methods

### 2.1 Subjects

A total of 79 patients with type 2 diabetic foot (DF) admitted to the Department of Endocrinology, Yiyang Central Hospital from June 2018 to March 2023 were selected (46 males, 33 females). Concurrently, 79 patients with type 2 diabetes admitted during the same period were selected as the control group (40 males, 39 females).

Inclusion criteria: (1). In accordance with the diagnostic criteria for type 2 diabetes outlined in the Chinese Guidelines for the Prevention and Treatment of Type 2 Diabetes (2020 Edition) (4): fasting blood glucose ≥ 7.0 mmol/L, blood glucose ≥ 11.1 mmol/L in 2 hours of random blood glucose or oral glucose tolerance test, and glycosylated hemoglobin ≥ 6.5%. For symptomatic cases of “three more than one less,” any blood sugar level meeting the standard is considered, and for asymptomatic cases of “three more than one less,” the blood sugar level should meet the above standard after two non-same-day tests. (2). According to the Chinese Expert Consensus on the Treatment of Diabetes Foot Amputation (Toe), diabetes foot amputation (toe) can be divided into large amputation and small amputation (toe) according to the plane of amputation (toe): Large amputation: Amputation above the ankle joint level due to the inability to alleviate severe disease through vascular reconstruction, drug control, or small amputation (toe). Small amputation (toe): Open or closed local amputation (toe) performed through partial vascular reconstruction or limb correction, clearing infected and necrotic tissues. Involves limited tissue removal, usually at the level of the ankle joint and below. (3). According to the 2007 Chinese Thyroid Disease Diagnosis and Treatment Guidelines of the Endocrinology Branch of the Chinese Medical Association, thyroid function status is defined as ESS: Decreased FT3, normal or decreased FT4, normal or decreased TSH.

Exclusion criteria: Individuals with other endocrine disorders, severe heart disease, liver and kidney disease, previous hyperthyroidism, hypothyroidism, subclinical hyperthyroidism, subclinical hypothyroidism, Hashimoto’s thyroiditis, family history of autoimmune thyroid disease, and currently taking thyroid disease related drugs or amiodarone, estrogen, androgen, or glucocorticoids. Individuals are taking common vitamin D2, ordinary vitamin D3 and active vitamin D.

### 2.2 Methods

Patients’ age, sex, and duration of diabetes, average length of hospital stay, amputation (toe) were gathered through the collection of medical history. The height and weight were measured using Omron Corporation’s Bluetooth instrument, and the body mass index (BMI) was subsequently calculated. Hospitalization days and amputation status were determined by reviewing case records.

Elbow vein blood was collected from study subjects on an empty stomach for at least 8 hours and sent to the laboratory for the measurement of fasting blood glucose and blood lipids (total cholesterol and triglycerides). In addition, C-reactive protein (CRP) was measured in the DF group as part of the clinical assessment of infection and inflammation. Serum 25OH -vitD and glycated hemoglobin were measured using a German Roche fully automatic electrochemiluminescence analyzer, which also assessed FT3, FT4, and TSH.

### 2.3 Control matching method

Individual matching was used to match each DF patient with one type 2 diabetes patient (without foot disease) from the same period. Matching variables included age, sex, and duration of diabetes. The matching was performed using the “case-control matching” function in SPSS 27.0. A total of 79 pairs (158 subjects) were successfully matched. There were no significant differences between the two groups in age, sex distribution, or disease duration (see **Table 1**).

**Table 1:** Demographic baseline.

Variable	T2DM(n=79)	DF(n=79)	P
Age(year)	57.90 ± 13.11	61.25 ± 11.01	0.08
Sex, n(%)			0.34
Male	40(50.6%)	46(58.2%)	
Female	39(49.4%)	33(41.8%)	

T2DM, type 2 diabetic; DF, Diabetic foot.

### 2.4 Sample size calculation

Sample size was estimated based on the prevalence of ESS as the primary outcome. According to previous literature, the prevalence of ESS in diabetic foot patients is approximately 25% (3). Assuming a power of 0.80 and a two−sided α of 0.05, the required sample size was calculated to be at least 62 patients per group using a two−proportion formula. Ultimately, 79 patients were included in each group, meeting this requirement. Due to the small subgroup sizes (major amputation, n=4; minor amputation, n=7), findings from amputation subgroup analyses are exploratory and warrant cautious interpretation.

### 2.5 Statistical methods

Data from all research subjects were collected and verified through double entry. Statistical analysis was performed using SPSS (version 27.0; IBM Corp., Armonk, NY, USA).

Quantitative data conforming to a normal distribution were represented as ( $\bar{\text{x}}$ ± s). Comparison between two groups was carried out using independent samples t-tests, while multiple group comparisons were performed using one-way ANOVA. *Post-hoc* pairwise comparisons were conducted using the LSD-t test only when the overall ANOVA was significant. Correlation analysis was conducted using Pearson correlation analysis. Count data were expressed in proportion or percentage, and intergroup comparisons were carried out using the x^2^ test. Binary logistic regression was used to identify risk factors for diabetic foot; continuous variables (e.g., FT3, age) were entered directly, and the categorical variable 25OH-vitD (deficiency <20 ng/mL, insufficiency 20-30 ng/mL, sufficiency >30 ng/mL) was entered with the “sufficiency” group as the reference via the “Categorical” dialog. ROC curves were used to evaluate predictive value: FT3 (continuous) was used directly as a test variable; for 25OH-vitD groups, they were rank-assigned (deficiency=1, insufficiency=2, sufficiency=3) and entered as a continuous variable; the combined predicted probability was obtained from the logistic regression model including both FT3 and 25OH-vitD categories. P value <0.05 was considered statistically significant.

## 3 Results

### 3.1 Comparison of general data, vitamin D, thyroid function, and ESS prevalence between type 2 diabetes and diabetic foot

The baseline demographic characteristics of the study participants are summarized in **Table 1**. The mean age of the DF group was 61.25 ± 11.01 years, compared with 57.90 ± 13.11 years in the T2DM group, and the difference was not statistically significant (P = 0.08). The DF group comprised 46 males (58.2%) and 33 females (41.8%), while the T2DM group comprised 40 males (50.6%) and 39 females (49.4%). No significant difference in sex distribution was observed between the two groups (P = 0.34). These results indicate that the two groups were well matched in terms of age and sex at baseline.

Independent samples t-test showed that serum 25OH-vitD and FT3 levels were significantly lower in DF patients than in type 2 diabetes patients (P<0.05). No significant differences were found in BMI, HbA1c, TC,TG,FBS,FT4, or TSH between the two groups (P>0.05) (**Table 2**).

**Table 2:** Comparison of general clinical data, vitamin D, and thyroid function between T2DM and DF (` $\bar{\text{x}}$ ± s, n).

Variable	T2DM(n=79)	DF(n=79)	t	P
BMI(kg/m^2^)	23.38 ± 1.81	23.75 ± 3.22	0.74	0.46
HbA1c(%)	9.78 ± 2.35	9.21 ± 2.82	-1.31	0.19
TG	2.64 ± 1.79	2.84 ± 2.73	-0.58	0.57
TC	4.52 ± 1.05	4.63 ± 1.32	-0.59	0.56
FBS	9.23 ± 3.17	8.52 ± 2.34	-1.63	0.11
VD(ng/mL)	21.16 ± 9.48	18.45 ± 6.42	-2.10	0.04
FT3(nmol/L)	4.08 ± 0.59	3.58 ± 0.99	-3.82	0.01
FT4(pmol/L)	15.42 ± 2.44	16.11 ± 3.29	1.50	0.14
TSH(mIU/L)	2.76 ± 1.86	2.59 ± 1.81	-0.61	0.54

VD, 25 hydroxyvitamin D; T2DM, type 2 diabetic; DF, Diabetic foot.

The x^2^ test showed that the prevalence of 25OH−vitD deficiency in DF patients was 70.9%, insufficiency 22.8%, and sufficiency 6.3%; in type 2 diabetes patients, these were 50.6%, 35.4%, and 13.9%, respectively. The prevalence of deficiency was significantly higher in the DF group (P<0.05) (**Table 3**).

**Table 3:** Comparison of serum 25OH-vitD levels between T2DM and DF ( $\bar{\text{x}}$ ± s).

Group	VD (ng/mL)			
	x ± s	<20(n,%)	20-30(n,%)	>30(n,%)
T2DM	21.16 ± 9.48	40 (50.6%)	28 (35.4%)	11 (13.9%)
DF	18.45 ± 6.42	56 (70.9%)	18 (22.8%)	5 (6.3%)

P=0.029 X2 = 7.091^a^ (Pearson chi-square) ;VD, 25 hydroxyvitamin D; T2DM, type 2 diabetic; DF, Diabetic foot.

The prevalence of ESS was 27.8% in DF patients and 3.8% in type 2 diabetes patients (P<0.05) (**Table 4**).

**Table 4:** Comparison of the prevalence of ESS between type 2 diabetes and type 2 diabetic foot.

	ESS (n,%)	NTF (n,%)
T2DM (n=79)	3 (3.8%)	76 (96.2%)
DF (n=79)	22 (27.8%)	57 (72.2%)

P = 0.01 X^2^ = 15.40 (Continuity correction) ;ESS, euthyroid sick syndrome; NTF, Normal thyroid function.

### 3.2 Multivariate analysis of risk factors for diabetic foot

Multivariate logistic regression (adjusted for age) showed that 25OH-vitD level was significantly associated with DF risk (overall P = 0.029). Using the sufficiency group (>30 ng/mL) as reference, the deficiency group (<20 ng/mL) had a significantly increased risk (OR = 3.539, 95%CI: 1.071–11.699, P = 0.038); the insufficiency group (20–30 ng/mL) showed no significant difference (OR = 1.556, P = 0.495). FT3 was an independent protective factor (OR = 0.493 per 1 pmol/L increase, 95%CI: 0.317–0.766, P = 0.002). Age was not significant (P = 0.306) (**Table 5**).

**Table 5:** Multifactorial logistic regression analysis of risk factors for diabetic foot in patients with type 2 diabetes mellitus.

Variable	B	SE	Wald χ2	P	OR	OR95% CI
Age	0.015	0.015	1.049	0.306	1.015	0.986 ~ 1.045
FT3	-0.708	0.225	9.913	0.002	0.493	0.317 ~ 0.766
VD(overall)			7.063	0.029		
VD(1)(deficiency vs. suffciency)	1.264	0.610	4.292	0.038	3.539	1.071~ 11.699
VD(2)(insuffciency vs. suffciency)	0.442	0.648	0.465	0.495	1.556	0.437~5.544

### 3.3 Predictive value of FT3, 25OH−vitD, and their combination for diabetic foot

**Figure 1:**
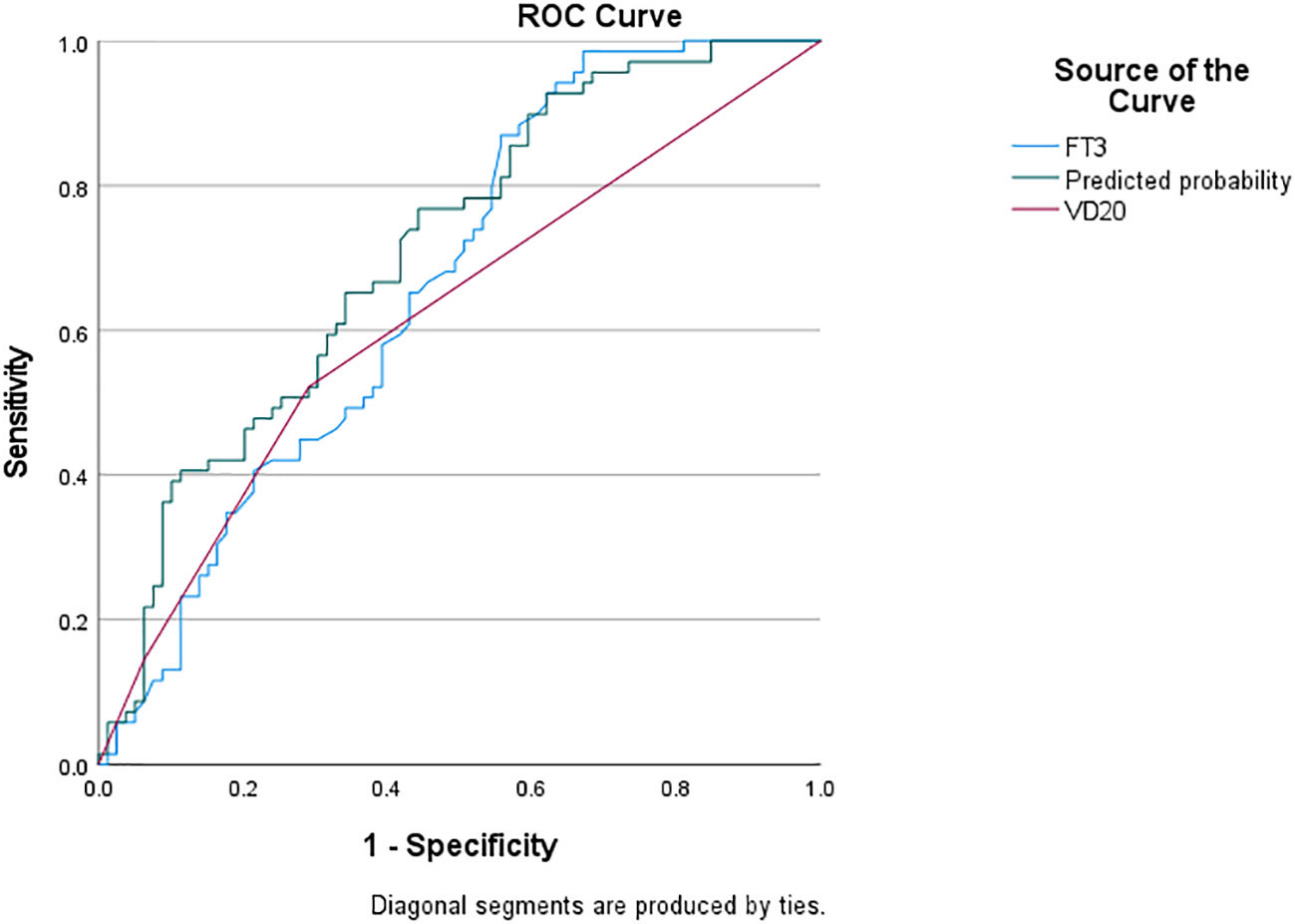
ROC curve.

ROC curve analysis showed that the AUC for 25OH-vitD (rank-assigned) alone was 0.620 (95%CI: 0.529–0.711, P = 0.012), for FT3 alone was 0.663 (95%CI: 0.576–0.750, P = 0.001), and for the combined predicted probability was 0.701 (95%CI: 0.618–0.785, P<0.001), indicating that the combined model had significantly better predictive performance than either single indicator (**Table 6**, **Figure 1**).

**Table 6:** Predictive value of FT3, 25OH-vitD and combined detection for type 2 diabetic foot.

Variable	AUC	*P*	95% CI
FT3(continuous)	0.663	0.001	0.576 ~0.750
VD(rank-assigned)	0.620	0.012	0.529 ~ 0.711
FT3+VD(combined)	0.701	0.001	0.618 ~ 0.785

### 3.4 Correlation of 25OH-vitD with general data and biochemical indicators in DF patients

Pearson correlation analysis showed that 25OH-vitD was negatively correlated with disease duration and CRP (P<0.05). No linear correlations were found with age, BMI, HbA1c, FT3, or length of hospital stay (P>0.05) (**Table 7**).

**Table 7:** The relationship between 25OH-vitD and general information and biochemical indicators in patients with type 2 diabetic foot.

Project	Age	BMI(kg/m^2^)	HbA1c(%)	CRP(mg/L)	FT3 (nmol/L)	DOD	LOHS
r	-0.16	0.04	-0.13	-0.25	0.01	-0.29	0.01
P	0.14	0.76	0.29	0.03	0.98	0.01	0.95

DOD, duration of diabetes ;LOHS, length of hospital stay.

### 3.5 Relationship between amputation and 25OH-vitD, thyroid function in DF patients

The ESS group had a significantly lower 25OH-vitD level than the euthyroid group (P<0.05). The amputation rate in the ESS group was 27.3%, significantly higher than that in the euthyroid group (8.8%, P = 0.03) (**Table 8**). The prevalence of ESS was 75.0% in the major amputation group, 42.9% in the minor amputation group, and 23.5% in the non−amputation group (P = 0.02) (**Table 9**).

**Table 8:** Comparison of 25OH-vitD levels and amputation rate between ESS and euthyroid patients in type 2 diabetic foot.

	n	VD ng/ml	Amputation rate
ESS	27	15.90 ± 7.50	27.3% (n=6)
NTF	52	19.20 ± 6.37	8.8% (n=5)
		t= -2.06	X2 = 4.53
P		0.04	0.03

NTF, Normal thyroid function; ESS, euthyroid sick syndrome; VD, 25 hydroxyvitamin D; (n,%).

**Table 9:** Comparison of ESS with different amputation status in type 2 diabetic foot.

	non-amputation (n,%)	minor amputation (n,%)	major amputation (n,%)
ESS	16 (23.5%)	3 (42.9%)	3 (75.0%)
NTF	52 (76.5%)	4 (57.1%)	1 (25.0%)

P = 0.02 X2 = 5.67 (Continuity correction); NTF, Normal thyroid function; ESS, euthyroid sick syndrome.

One-way ANOVA showed significant differences in CRP and TSH among the non-amputation, minor amputation, and major amputation groups (P<0.05). Post−hoc analysis (LSD-t test) indicated that patients with major amputation had significantly higher CRP and TSH levels than non-amputation patients. Although FT3 and 25OH-vitD showed a decreasing trend across the three groups (FT3: P = 0.08, 25OH−vitD: P = 0.13), the differences did not reach statistical significance, possibly due to the small sample size (**Table 10**).

**Table 10:** The incidence of amputation and the changes of 25OH-vitD, thyroid function and clinical data in type 2 diabetic foot ( $\bar{\text{x}}$ ± s).

	Non-amputation(n=68)	Minor amputation(n=7)	Major amputation(n=4)	F	P
Age(year)	61.26 ± 11.37	60.00 ± 10.40	63.25 ± 6.08	0.11	0.89
BMI(kg/m^2^)	23.77 ± 3.18	23.92 ± 6.04	23.53 ± 1.80	0.22	0.80
DOD(year)	10.71 ± 7.51	9.00 ± 7.84	16.25 ± 7.50	1.25	0.29
HbA1c(%)	9.14 ± 2.73	8.03 ± 3.02	12.00 ± 2.85	2.60	0.08
CRP(mg/L)	44.24 ± 44.97	24.25 ± 22.96	95.65 ± 52.16*	3.45	0.04
VD(ng/mL)	18.75 ± 6.33	19.19 ± 7.86	12.21 ± 0.50	2.06	0.13
LOHS(day)	17.47 ± 12.39	23.43 ± 4.86	28.00 ± 15.09	2.06	0.13
FT3(nmol/L)	3.63 ± 0.97	3.67 ± 1.21	2.52 ± 0.50	2.49	0.08
FT4(pmol/L)	16.03 ± 3.31	16.13 ± 3.59	17.40 ± 3.03	0.32	0.72
TSH(mIU/L)	2.31 ± 1.61	4.09 ± 2.47	4.59 ± 1.58*	6.48	0.01

DOD, duration of diabetes; LOHS, length of hospital stay: *One-way ANOVA showed significant differences only in CRP and TSH among the three groups (P < 0.05). *Post-hoc* analysis (LSD-t test): *P < 0.05 compared to the non-amputation group.

## Discussion

Type 2 diabetes mellitus (T2DM) constitutes a major global public health challenge, and its continuously increasing prevalence has led to a sustained rise in the risk of chronic complications. As one of the complications with the most difficult treatment and the poorest prognosis, type 2 diabetic foot disease imposes a heavy burden on society and families. Currently, the severity assessment of diabetic foot (DF) mainly relies on clinical systems such as the Wagner classification, which fails to fully integrate systemic metabolic and endocrine indicators. The core finding of this preliminary study is that patients with type 2 diabetic foot not only present with lower levels of 25OH-vitamin D (25OH-vitD) and free triiodothyronine (FT3), as well as higher prevalence of vitamin D deficiency and euthyroid sick syndrome (ESS), but more importantly, the area under the curve (AUC) of combined assessment of FT3 and 25OH-vitD for predicting diabetic foot is higher than that of either single indicator, which introduces a “multiple endocrine axes” perspective for DF assessment.

### Scientific rationale for combined assessment of vitamin D and thyroid function

Most previous studies have separately explored the independent effects of vitamin D and thyroid function on diabetic foot (2, 3, 5–8), while few studies have jointly assessed these two pathways. Our choice of combined assessment is based on the following evidence: First, vitamin D is a novel immunoregulatory hormone that extensively affects a variety of endocrine and autoimmune diseases including diabetes mellitus and thyroid diseases (9). Its receptor (VDR) belongs to the steroid/thyroid hormone receptor superfamily, shares structural homology with thyroid hormone receptors (10), and is widely distributed in systemic tissues such as the pancreas, thyroid gland and skin (11–13). Second, the occurrence and progression of T2DM involve complex endocrine crosstalk among multiple organs including bone, vitamin D regulatory axis and thyroid axis, and vitamin D is a key component of this integrated network (14). Third, both vitamin D and thyroid function are independently associated with diabetic microvascular complications and impaired wound healing (2, 3). These mechanisms provide a theoretical basis for the “multiple endocrine axes” combined assessment proposed in this study.

### Vitamin D deficiency and diabetic foot

The high prevalence of vitamin D deficiency observed in DF patients in this study is consistent with the results from a large-scale study of Chinese hospitalized T2DM patients (5, 15). An umbrella review encompassing 8 meta-analyses confirmed that severe vitamin D deficiency can increase the risk of diabetic foot ulceration (DFU) by at least 1.82-fold (16). Tang et al. demonstrated that vitamin D deficiency is an independent risk factor for diabetic foot osteomyelitis (DFO) (OR = 3.05) (17).

while our study shows that this risk can be as high as 3.5-fold, which further strengthens the strong correlation between the two factors. The negative correlation between 25OH-vitD and C-reactive protein (CRP) observed in our study supports the hypothesis that vitamin D level may help assess the inflammatory status of diabetic foot, which is consistent with the finding that vitamin D level is negatively correlated with Armstrong classification (18).

At the mechanistic level, existing basic studies have confirmed that vitamin D can promote the healing of diabetic foot ulcers. Gonzalez-Curiel et al. (19)demonstrated that the active form of vitamin D can induce keratinocytes from DFU patients to produce antimicrobial peptides LL-37 and HBD-2, enhancing local antimicrobial defense. Trujillo et al. (20) further proved that calcitriol can upregulate the expression of pro-angiogenic molecules such as vascular endothelial growth factor (VEGF) to improve the healing of ischemic tissues. In addition, López-López et al. (21) found that calcitriol can regulate the MMP/TIMP balance and promote the transformation of chronic wound environment towards a healing-prone state. These cellular and molecular evidences provide strong support for the clinical observations of this study.

Regarding amputation outcomes, our small-sample exploratory analysis shows that lower vitamin D level is associated with more severe amputation outcomes, which is consistent with the conclusion that 25-(OH)D is an important predictor of minor amputation (15). Further,lower vitamin D level is independently associated with impaired DF wound healing, and vitamin D is an independent protective factor for DFU (22, 23). Our observational findings are further corroborated by a recent key interventional trial which demonstrated in a randomized controlled trial that daily vitamin D supplementation in patients with diabetic foot ulcers significantly reduced the infection rate and promoted wound healing, providing high-quality causal evidence for the clinical benefits of vitamin D supplementation (24). These findings collectively suggest that routine testing and appropriate vitamin D supplementation are necessary for DF patients.

### Thyroid dysfunction and diabetic foot

This study observed that DF patients have lower FT3 levels, and the prevalence of ESS (27.8%) is significantly higher than that in patients without foot disease (3.8%), which is consistent with the research results reported by Zhang et al. from Ruijin Hospital, Shanghai (3).

Our findings are further supported by a recent comparative study which demonstrated that DFU patients exhibit significantly more pronounced metabolic disturbances—including alterations in thyroid function—compared with diabetic patients without foot ulcers, even when glycemic control is comparable (25). This suggests that thyroid dysfunction in DF is not merely a consequence of hyperglycemia but is part of a broader pattern of adverse metabolic characteristics associated with diabetic foot disease.A recent retrospective cohort study of 726 DFU patients demonstrated that low FT3 and low FT3/FT4 ratio are independent risk factors for all-cause mortality in DFU patients (26).

This association may be related to multiple pathophysiological mechanisms: long-term hyperglycemia can inhibit the conversion of thyroxine (T4) to triiodothyronine (T3) (27); as foot disease progresses, persistent chronic inflammatory response and tissue hypoxia reduce thyroid hormone synthesis; in addition, thyroid-stimulating hormone (TSH) level is also directly associated with insulin resistance and oxidative stress induced by advanced glycation end products (AGEs) (28, 29). A study by Nong found that FT3 level decreases with the increase of Wagner grade (30), and our results also observed that FT3 level shows a downward trend while TSH level is higher in the major amputation group, which further verifies this pattern.

An important novel finding of this study is that the prevalence of ESS increases in a stepwise manner with the severity of amputation, and the prevalence in the major amputation group reaches as high as 75%. A Chinese clinical study on elderly DF amputation patients complicated with ESS also showed that inflammatory markers are negatively correlated with FT3 level, and ESS is a characteristic manifestation associated with poor outcomes in critically ill patients (3, 27). This indicates that ESS is not merely a laboratory abnormality, but a clinically significant comorbid condition that reflects the severity of DF. This finding highlights the potential value of routine thyroid function screening for DF patients to identify populations at high risk of amputation.

### Innovation and clinical value of combined assessment

To the best of our knowledge, this study is the first to specifically investigate the predictive value of combined assessment of vitamin D and thyroid function for DF. Our key finding is that the AUC of combined assessment is higher than that of either single indicator, suggesting that these two pathways may have synergistic value in the assessment of DF. This not only introduces a “multiple endocrine axes” perspective beyond traditional wound grading for the clinical assessment of DF, but if validated by larger-scale studies, this method may provide clinicians with a convenient and objective auxiliary tool for risk stratification in the future.

This study has several limitations. First, and most importantly, this is a single-center pilot study with a limited sample size. The extremely small amputation subgroups (major amputation, n = 4; minor amputation, n = 7) severely limit the statistical power. Therefore, these findings should be considered exploratory and interpreted with caution. Second, as a single-center retrospective study, there may be selection bias and unmeasured confounding factors. Importantly, detailed information on several key potential confounders—including concomitant medications (e.g., metformin, insulin, statins), lifestyle factors (e.g., smoking status, dietary vitamin D intake, daily sun exposure), socioeconomic status, and patients’ level of disease knowledge—was not systematically recorded in the medical records and could not be retrieved for analysis. These unmeasured factors may significantly influence vitamin D levels, thyroid function, and diabetic foot outcomes, and therefore the observed associations are subject to residual confounding. Third, the cross−sectional design precludes causal inference. Fourth, we did not assess the effect of vitamin D supplementation on clinical outcomes, nor did we explore the correlation between vitamin D and other autoimmune thyroid diseases. Given these important limitations, the present findings should be regarded as preliminary. Future multicenter prospective studies with larger sample sizes, comprehensive collection of potential confounders, and extend our observations and to investigate the effects of vitamin D intervention on inflammatory status, thyroid function, ulcer healing, and overall prognosis in diabetic foot patients.

## Conclusions

In summary, this preliminary study demonstrates that patients with type 2 diabetic foot have higher prevalence of 25OH-vitD deficiency and ESS, and severe vitamin D deficiency and ESS are associated with poor prognosis and increased amputation risk. This highlights the clinical value of routine testing of thyroid function and 25OH-vitD for patients with type 2 diabetic foot. The combined assessment perspective proposed in this study may provide a convenient and objective auxiliary tool for clinicians. Given the preliminary nature of this study, multi-center prospective studies with larger sample sizes are needed in the future to comprehensively collect potential confounding factors and incorporate additional nutritional markers, so as to validate these findings and explore their impact on DF outcomes.

## Data Availability

The datasets used or analyzed during the current study are available from the corresponding author on reasonable request.
